# Fermented Vinegars from Apple Peels, Raspberries, Rosehips, Lavender, Mint, and Rose Petals: The Composition, Antioxidant Power, and Genoprotective Abilities in Comparison to Acetic Macerates, Decoctions, and Tinctures

**DOI:** 10.3390/antiox9111121

**Published:** 2020-11-13

**Authors:** Małgorzata Kalemba-Drożdż, Inga Kwiecień, Agnieszka Szewczyk, Agnieszka Cierniak, Agata Grzywacz-Kisielewska

**Affiliations:** 1 Department of Biochemistry, Faculty of Medicine and Health Sciences, Andrzej Frycz Modrzewski Krakow University, Gustaw Herling-Grudziński St. 1, 30-705 Krakow, Poland; acierniak@afm.edu.pl (A.C.); agrzywacz-kisielewska@afm.edu.pl (A.G.-K.); 2 Department of Pharmaceutical Botany, Medical College, Jagiellonian University, Medyczna St. 9, 30-688 Krakow, Poland; inga.kwiecien@uj.edu.pl (I.K.); agnieszka.szewczyk@uj.edu.pl (A.S.)

**Keywords:** polyphenols, antioxidants, fermented vinegar, genoprotection, chemoprevention, DNA damage, *Rosa canina*, *Rosa rugosa*, plant extracts

## Abstract

Acetic fermentation is a method for processing plant material which has been known since antiquity. Balsamic and apple cider vinegars are investigated as antibacterial, anti-obesity, and anti-diabetic remedies. However, there is little information about vinegars fermented from aromatic herbs and edible plants. The aim of this study was to compare extracts used for culinary and medicinal purposes according to their composition, antioxidant power, and genoprotective properties. Fermented vinegars, acetic macerates, decoctions, and tinctures in 70% ethanol from raspberries, apple peels, rosehips, lavender, mint, and rose petals were prepared. Polyphenols, ascorbate, carotenoid concentrations, and antioxidant power were analyzed. The polyphenols were identified using HPLC (high-performance liquid chromatography). The genoprotective properties were measured using a comet assay on lymphocytes. Fermented vinegars were poorest in phytochemicals in comparison to tinctures, decoctions, or acetic macerates, although they contained the highest concentration of metal ions. The antioxidant abilities were correlated to the phenolic content of extract. None of the extracts induced DNA damages into lymphocytes. The rosehip and rose petal extracts revealed the highest genoprotective abilities, while mint and apple fermented vinegars and decoctions had the lowest. Fermented vinegars are not a rich source of phytochemicals and they show weak genoprotective abilities, but, in increasing demand for antioxidants, any form of phytochemical sources is an added-value in diet.

## 1. Introduction

In the temperate climate zone of the middle-latitudes and subpolars, where fresh fruit and herbs are not available during the whole year, seasonal plant products are traditionally processed into preserves, such as jams, syrups, compotes, pickles, and tinctures. Traditional methods of extracting the active ingredients from fruits and herbs are mainly based on macerating fruits and herbs in ethanol (tinctures/intracts) or conserving them with large amounts of sugar. However, certain groups of people, such as children, pregnant women, diabetes, people suffering from gout or pancreatitis, and people taking certain medicines should restrict their alcohol and sugar intake. An alternative means of processing fruits and herbs for culinary and health-promoting purposes, which is currently greatly gaining in popularity, is vinegar fermentation. Perhaps naturally fermented vinegars may be an additional source of antioxidants in the diet, especially in the winter, while not being subject to the restrictions associated with products containing sugar or alcohol.

Vinegar fermentation from raw fruit juices or plant material in water with sugar proceeds in two stages [1]. At the first stage, the yeasts *Saccharomyces* convert sugars (glucose, fructose, and saccharose—added or naturally occurring) into ethanol during alcoholic fermentation. The second stage is performed by the acetic acid bacteria *Acetobacter*, which oxidize ethanol to carbon dioxide and produce acetic acid during “oxidative fermentation”, also known as pseudo-fermentation, as typical fermentation is an anaerobic process. A greater variety of microorganism species is usually involved in home fermentations than in industrial processes where controlled starter cultures are used [2]. Some species of acetic acid bacteria, such as *Acetobacter xylinum*, are able to produce cellulose, then a mother of vinegar, also known as “mycoderma aceti”, may appear on the surface of the vinegar.

Intensive research is currently being conducted on the health benefits of apple cider vinegar and balsamic vinegar. First of all, it has been shown that they are the source of phenolic compounds and they have antioxidant abilities [3,4,5]. Moreover, apple cider and balsamic vinegars reveal antibacterial properties [6,7] and they reduce the risk of hyperlipidemia and obesity and attenuate the oxidative stress [8,9,10]. There are reports that consuming apple cider vinegar diluted with water lowers blood glucose and slows gastric emptying rates [11,12,13]. The anti-glycemic effect is probably due to delayed absorption and digestion of polysaccharides [1]. The effect of vinegars on the digestion rate probably also causes changes in the absorption of fat components, which has been found in studies on rats [8,11,14]. However, there are few reports on the properties of vinegars fermented from other fruits and herbs, and in the light of publications on the health benefits of apple cider vinegar as the source of antioxidants, we considered that it would be interesting and valuable, from a practical point of view, to investigate the composition, antioxidative, and genoprotective properties of fermented vinegars.

The aim of this research was to analyze vinegars fermented from fruits and herbs, according to the phytochemical composition, the antioxidant properties, and the ability to prevent DNA damage. The study compared the fermented vinegars to other types of extracts traditionally used in cookery and medicine: acetic macerates, decoctions, and tinctures prepared from the same plants.

## 2. Materials and Methods

### 2.1. Reagents

The following reagents were obtained from Merck (Sigma-Aldrich Co., St Louis, MO, USA): Histopaque 1077; trypsin; ethylenediaminetetraacetic acid (EDTA); ethidium bromide; 4-Amino-2,5-diethoxybenzanilide diazotated zinc double salt (FBBB—Fast Blue BB reagent); 3-(4,5-dimethylthiazol-2-yl)-2,5-diphenyltetrazolium bromide (MTT); hydrogen peroxide; dimethyl sufloxide (DMSO); 2-[4-(2,4,4-trimethylpentan-2-yl)phenoxy]ethanol (Triton X-100); 2-Amino-2-(hydroxymethyl)propane-1,3-diol (Trizma Base); and 2′,7′-dichlorofluorescein diacetate. The other reagents were: 2,4,6-Tris(2-pyridyl)-s-triazine (TPTZ)—Fluka Analytical, Bucha, Switzerland; agarose-normal melting point (NMPA), agarose-low melting point (LMPA)—Gibco BRL, Karlsruhe, Germany; Fetal Bovine Serum (FBS)—Lonza, Basel, Switzerland; penicillin; streptomycin—Biomed, Lublin, Poland; *propidium* iodide—MP Biomedicals, Eschwege, Germany; RPMI-1640—Cytogen GmbH, Wetzlar, Germany; chlorophorm; ethanol; methanol; AlCl_3_; HCl; KCl; NaCl; NaNO_2_; NaOH; phosphate buffer saline (PBS) without (*w*/*o*) Ca^2+^ and Mg^2+^; sodium acetate trihydrate; ferrous sulfate heptahydrate—Avantor Performance Materials, Gliwice, Poland; and iron (III) chloride hexahydrate—PARK Scientific, Northampton, UK.

The standards for high-performance liquid chromatography (HPLC) of anthocyaninswere: callistephin chloride; cyanidin-3-glucoside; cyanidin chloride; idaein chloride; keracyanin chloride; kuromanin chloride; malvidin chloride; malvidin-3-*O*-galactoside chloride; malvin chloride; oenin chloride; pelargonidin chloride (Sigma Aldrich Co., St Louis, MO, USA). The standards for HPLC of flavonoids and phenolic acids were: apigenin; baicalein; chrysin; cynaroside; luteolin; myricetin; naringoside; quercetin; quercitrin; robinin; rutoside; scutellarein; trifolin; vitexin; wogonoside; caffeic acid; cinnamic acid; chlorogenic acid; m-coumaric acid; o-coumaric acid; p-coumaric acid; 3;4-dihydroxyphenylacetic acid; ellagic acid; ferulic acid; isoferulic acid; gallic acid; gentisic acid; hydrocaffeic acid; p-hydroxybenzoic acid; neochlorogenic acid; phenylacetic acid; 3-hydroxyphenylacetic acid; 4-*O*-feruloylquinic acid; protocatechuic acid; rosmarinic acid; salicylic acid; sinapic acid; syringic acid; vanillic acid; baicalin; diosmetin; kaempferol; scutellarin; wogonin; verbascoside; isoverbascoside; isoquercetin (ChromaDex, Irvine, CA, USA); benzoic acid (Merck, Darmstadt, Germany); apigetrin; hyperoside; isorhamnetin; isorhamnetin 3-rhamnoside; kaempferol 3-rhamnoside; kaempferol 7-rhamnoside; quercetin-7-*O*-beta-glucopyranoside; populnin; rhamnetin (isolated in the Department of Pharmacognosy, Medical University of Gdańsk, Gdańsk, Poland); isochlorogenic acid; cryptochlorogenic acid; caftaric acid; p-coumaric acid methyl ester; oroxylin A; skullcapflavone II; quercetin-3-*O*-glucoside-7-*O*-rhamnoside; guaijaverin; naringenin 7-*O*-rutinoside; kaempferol 3-*O*-rhamninoside; miquelianin; kaempferol 3-neohesperidoside; apigenin-7-glucuronide; apigenin 5-*O*-beta-glucopyranoside; apigenin 4′-rhamnoside; kaempferol 4′-*O*-beta-D-glucopyranoside; quercetin-*O*-arabinofuranoside; catechin; epicatechin; epigallocatechin; epigallocatechin gallate; epicatechin gallate (ChemFaces Biochemical Co., Wuhan, China.

Raspberries, mint, and lavender were commercially available in local shops as they had been harvested during the summer season while rosehips and apples were harvested in the autumn. Food vinegar (8% acetic acid) was bought in a local shop in Krakow, Poland. Rose petals (*Rosa rugosa*) were harvested in the wild regions of Małopolska (Poland).

### 2.2. Plant Extracts

Four types of extracts were analyzed, namely: fermented vinegars, acetic macerates, decoctions, and tinctures in 70% ethanol prepared from raspberries (*Rubus idaeus*), the skins peeled of apples (*Malus domestica*), the flesh of rosehips (*Rosa canina*), lavender flowers (*Lavandula angustifolia*), mint leaves (*Mentha spicata*), and rose petals (*Rosa rugosa*). The fruit extracts were prepared using 30% of plant material input, and the herbal extracts contained 2% of plant material input. The amount of plant material was chosen according to the observations of herbalists experienced in vinegar fermentation, adapting the intuitive volumetric method into weight standards. The extracts were prepared three times, each time using a single batch of plant material for all types of extracts.

#### 2.2.1. Fermented Vinegars

The fermented vinegars were prepared from 30 g of fruits or 2 g of herbs, and 8 g of saccharose in a suspension of a total of 100 g, as is practiced in Polish, Ukrainian, and Russian homes. The suspensions were kept in covered but not closed bottles and mixed twice a day for 4 weeks. When the acetic fermentation had finished, the vinegar was merged from above the sediment. The vinegars were prepared three times for each of the fruits and herbs.

#### 2.2.2. Acetic Macerates

Eight percent acetic acid (food vinegar) was poured over 30 g of fruits or 2 g of herbs in a suspension of a total of 100 g and kept in closed bottles at room temperature for 4 weeks. Then, the macerate was merged from above the plant sediment. The macerates were prepared three times for each of the fruits and herbs.

#### 2.2.3. Decoctions

Water was poured over 30 g of fruits or 2 g of herbs to obtain a total amount of 100 g and incubated in closed bottles in a water bath at 100 °C for 25 min. The decoctions were prepared three times for each of the fruits and herbs. The extracts were stored at room temperature for 4 weeks.

#### 2.2.4. Tinctures

Seventy percent ethanol was poured over 30 g of fruits or 2 g of herbs to obtain a total weight of 100 g and kept in closed bottles at room temperature for 4 weeks. The ethanolic extracts were prepared three times for each of the fruits and herbs.

Once the extracts had been prepared, the samples were centrifuged at 5000 rpm for 10 min and the supernatant was collected and stored at −80 °C. In the phytochemical experiments, different types of blank samples were used for different types of extracts; for fermented vinegars and decoctions: water, for acetic macerates: food vinegar, and for the tinctures: 70% ethanol.

### 2.3. Total Polyphenol Content—Fast Blue BB Assay

The concentration of polyphenols was analyzed with Fast Blue BB assay (FBBB) [15] which ensures the lack of interference with ascorbic acid; 2 mL of sample was mixed for 1 min with 0.2 mL of 0.1% Fast Blue BB reagent (Sigma-Aldrich Co., St Louis, MO, USA). Then, 0.2 mL of 5% NaOH was added. The samples were incubated at room temperature for 90 min. The absorbance was measured with a LEDetect spectrophotometer (Labexim, Lengau, Austria) at 420 nm. Each sample was analyzed in triplicate. The results were expressed as quercetin equivalent [mg/mL].

### 2.4. Polyphenol Identification with HPLC

The extracts were analyzed using a HPLC system with Diode-Array Detector (DAD) (Elite LaChrome, L-2000 series, Hitachi, Tokyo, Japan) equipped with a Purospher RP-18e analytical column (4 × 250 mm, 5 μm, Merck, Darmstadt, Germany) at 25 °C, using a method described previously [16].

The mobile phase consisted of methanol (A) and 0.5% (*v*/*v*) acetic acid (B). The flow rate was set at 1.0 mL min^−1^ with an injection volume of 10 μL. The gradient elution scheme used was: (A/B ratio) 20:80%, *t*  =  0–20 min; 30–70%, *t*  =  35 min; 60–40%, *t*  =  60 min; 100–0%, *t*  =  70–75 min; 20–80%, *t*  =  80–90 min. Polyphenols were estimated at 254 nm (recording range of 200–400 nm). Quantification analyses were based on a comparison with the standard compounds listed in Section 2.1. The reagents were: flavonoids (29), catechins (5), and phenolic acids (25). Each extract was analyzed in triplicate.

### 2.5. HPLC Analysis of Anthocyanins

The extracts were filtered through a 0.2 µm filter and analyzed using the HPLC-DAD system. RP-HPLC (*reverse phase* high-performance liquid chromatography)analysis was performed according to a procedure described elsewhere [17] with our modification for a Merck–Hitachi liquid chromatograph (LaChrom Elite, L-2000 series, Hitachi, Tokyo, Japan) equipped with a DAD detector L-2455 (Hitachi, Tokyo, Japan) and Purospher RP-18e (250 × 4 mm/5 mm) column. The analysis were performed at 25 °C, with a mobile phase consisting of A—acetonitryle and B—0.1% formic acid. The gradient was as follows: 95–80% B for 0–15 min; 80–70% B for 15–20 min; 70% B for 20–25 min; 70–10% B for 25–30 min; 10% B for 30–35 min; 10–95% B for 35–45 min; and 95% B for 45–55 min at a flow rate of 1 mL min^−1^, λ = 520 nm. Each extract was run in triplicate. The identification of anthocyanins was performed by comparing the retention times of the peaks with the authentic reference compounds and co-chromatography with the standards. The anthocyanins were quantified by measuring the peak area with reference to the standard curve derived from five concentrations (0.03125 to 0.5 mg mL^−1^) [17].

### 2.6. Vitamin C Content with Tillman’s Method

Tillman’s method is based on the reduction of 2,6-dichlorophenolinodophenol (DCPIP, Tillman’s dye) by ascorbic acid; however, the modification was introduced with usage of an organic solvent (chloroform) due to the intensive coloration of fruit extracts [18]. Each sample was analyzed in triplicate. The 1-mg/mL ascorbic acid solution was used as a standard.

### 2.7. Carotenoid Content

The carotenoid concentration was obtained using the spectrophotometric method [19] for the 5 predominant carotenoid species, namely beta-carotene, zeaxanthin, lycopene, lutein, and beta-cryptoxanthin. The samples were evaporated using a vacuum concentrator CentriVap (Labconco, Kansas City, MI, USA) at 1725 rpm, 4 °C and the solids were diluted in acetone. The absorbance was measured with the LEDetect spectrophotometer (Labexim, Lengau, Austria) at 450 nm. The samples were analyzed in triplicate.

### 2.8. Metal Content

The metal content of the extracts was studied in the Accredited Hydrogeochemical Laboratory at the AGH University of Science and Technology (Krakow, Poland) using the inductively coupled plasma mass spectrometry (ICP-MS) method on an ELAN 6100 spectrometer (Perkin Elmer, Waltham, MA, USA). The calcium, magnesium, iron, zinc, aluminum, arsenium, cadmium, chromium, mercury, nickel, and lead contents were analyzed in accordance with the norm: PN-EN ISO 17294-2: 2016-11.

### 2.9. Ferric Reducing Antioxidant Power (FRAP) Assay

The FRAP assay [20] was adapted with minor modifications to evaluate the reducing power of the plant extracts. The absorbance was measured at 590 nm using the LEDetect spectrophotometer (Labexim, Lengau, Austria); 0.1% vitamin C and 5 mM quercetin were used as a standard antioxidant solution. Each sample was analyzed in triplicate. The results were expressed as the equivalent of Fe^2+^ ion concentration.

### 2.10. Cell Culture and Treatment

Human peripheral blood lymphocytes (PBML) were isolated from heparinized blood of two white, healthy donors aged 25 and 39 (the Regional Blood Donation and Transfusion Center, Kraków, Poland). The gradient centrifugation method with Histopaque 1077 (Merck-Sigma-Aldrich Co., St Louis, MO, USA) was conducted according to the manufacturer’s protocol. The lymphocytes were stored frozen until the experiment at −80 °C in 50% fetal bovine serum (FBS), 40% RPMI-1640, and 10% DMSO.

In each experiment, the cells were thawed in 50% FBS in RPMI-1640 and centrifuged at 1200 rpm at 4 °C for 5 min. Subsequently, the lymphocytes were suspended in RPMI-1640 with 10% FBS in a 96-well plate at a density of 1 × 10^4^ cells per well and incubated at 37 °C in 5% CO_2_ for 30 min [21].To evaluate the genotoxicity/genoprotection, the lymphocytes were treated with plant extracts for 1 h and 25 µM H_2_O_2_ for 5 min. The untreated cells were used as the negative control sample. The extracts were used in 0.1% dilution, as it had been established previously [18,22,23] that this is a concentration of flavonoid in vitro culture that resembles the flavonoid level in serum obtained after dietary ingestion of 60 mL of fruit juice [18,22,23]. All the experiments in cells were conducted in lymphocytes of the two donors independently and in duplicate for each extract.

### 2.11. Evaluation of Cell Viability with MTT Assay

In order to measure cell viability in the presence of plant extracts, the MTT staining method was employed [24]. Nicotinamide adenine dinucleotide or nicotinamide adenine dinucleotide phosphate (NAD(P)H)-dependent cellular oxidoreductase enzymes may reduce the tetrazolium dye (MTT) 3-(4,5-dimethylthiazol-2-yl)-2,5-diphenyltetrazolium bromide to its insoluble formazan. The activity of enzymes reflects the number of viable cells. The cells were seeded at 2 × 10^4^ cells/well in 96-well plates for 1 h with 0.1% extract added to the respective wells. MTT was added to the cells at a concentration of 0.5 mg/mL and incubated at 37 °C for 2.5 h. Then, 150 μL of isopropanol was added, shaken on a shaker for 15 min, and then, the absorbance was measured with an LEDetect spectrophotometer (Labexim, Lengau, Austria) at 590 nm.

### 2.12. Evaluation of DNA Damage with Comet Assay

The comet assay bases on a migration of nucleic DNA through an electrophoresis gel and the pattern revealed in a fluorescent microscope resembles a comet. The fluorescence intensity of the comet tail depends on the amount of DNA damage in the nucleus. The comet assay was performed under alkaline conditions, as described previously [18]. The propidium iodine staining was used for comet visualization in a fluorescent microscope Olympus IX50 (Olympus, Tokio, Japan) equipped with a 515–560-nm excitation filter and a 590-nm barrier filter at a magnification of 200×. Using the software COMET ASSAY IV trial version (Instem, Staffordshire, UK), 100 randomly selected cells were scored per sample. All samples were analyzed in duplicates. The results of the two independent experiments on the lymphocytes of the two donors were expressed as the Tail DNA Content (TDC).

### 2.13. Statistical Analysis

The statistical analysis was carried out using the software Statistica 9 (StatSoft-Tibco, Palo Alto, CA, USA). The results obtained from the lymphocytes donated by the donors did not differ significantly and they are expressed as the mean from all the experiments. The statistical significance of the differences between the experimental conditions in the comet assay was assessed via a one-way ANOVA test under the condition of positive homogeneity of variance in Levene’s test. The posteriori Tukey’s test was performed. The correlation analysis was carried out with linear regression and the strength of correlation was established via Pearson’s correlation factors. According to the large differences between the types of extract from different plant material, the statistical significance was evaluated using the Chi square test. A *p* probability of null hypothesis of 0.05 was considered as the cutoff for significance.

## 3. Results

### 3.1. Composition

**Figure 1:**
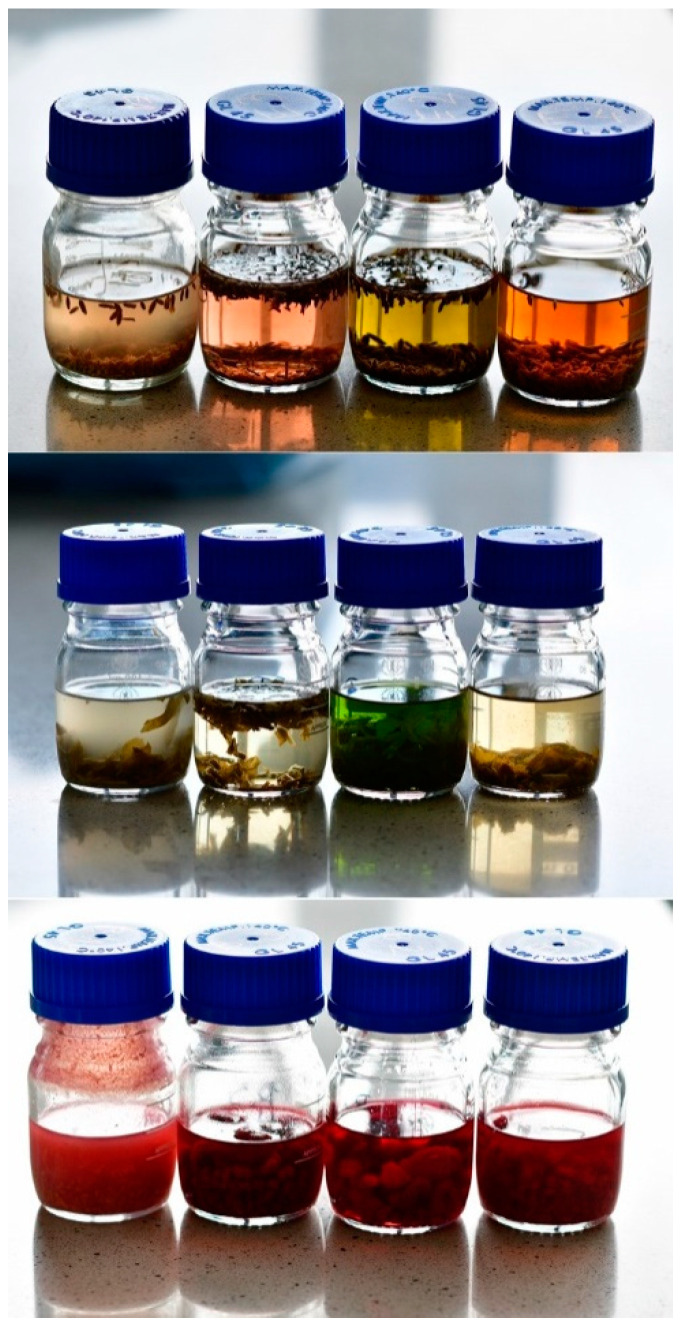
The plant extracts from lavender (**top**), mint (**middle**), and raspberry (**bottom**); from left: fermented vinegars, acetic macerates, tinctures, and decoctions.

The types of extract differed in color and translucency as in the examples shown in Figure 1.

The types of extract differed in composition (Table 1). Relatively high amounts of vitamin C was found in all rosehip extracts. In the raspberry extracts, ascorbic acid was detected in very small amounts except in the decoction, where ascorbic acid was not found. None of the extracts from apple peels, mint leaves, lavender flowers, and rose petals contained detectable amounts of vitamin C.

The trace amounts of carotenoids were present in the extracts. The fermented vinegars had slightly higher pH than the acetic macerates, but the difference was not significant (Table 1).

It was observed that all the rosehip extracts had the highest concentration of polyphenols. The rose petal extracts and raspberry tincture were characterized by a high polyphenol content. The lowest concentration of polyphenols was found in all the mint extracts. The total phenolic content was the highest in tinctures in the case of apple peels, raspberries, and rose petals as well as in the decoctions made from lavender and rosehips. The tinctures were found to have the highest phenolic content while the fermented vinegars were characterized by the lowest phenolic content (*χ*^2^ = 9.333; *df* = 3; *p* = 0.025; rank test *χ*^2^ = 6.171; *df* = 3, *p* = 0.104). The polyphenol concentrations measured via the spectrophotometric method and HPLC were slightly correlated (*y* = 0.1276*x* + 21.418; *R*^2^ = 0.3297; *p* = 0.05).

The HPLC analysis of the anthocyanins revealed that these compounds were present only in the apple peels and raspberry extracts. Kuromanin was found in the apple peel extracts, except in fermented vinegar (Table 2). Idaein and keracyanin, on the other hand, was detected in raspberry extracts. The tinctures from these plants were the type of extract characterized by the highest amounts of anthocyanins.

The general characteristic of different types of extracts revealed that the highest flavonoids concentration was found in tinctures and the lowest in fermented vinegars (Table 2).

The chromatography of flavonoids and phenolic acids confirmed that, in the case of the apple peel extracts, the highest amount of phenolic compounds was present in the tincture and lowest in the fermented vinegar (Table 2). Among the flavonoids, quercetin 3-*O*-glucuronide, quercetin 3-*O*-ramnoside, and catechin were present in the highest concentration. The situation was similar in the case of the raspberry extracts: fermented vinegars were characterized by the lowest concentration and the smallest diversity of flavonoids. In the raspberry extracts, the dominant polyphenol was elagic acid. The dominant polyphenol of the rosehip extracts was catechin, although the reship decoction was much richer in elagic acid than other types of rosal extracts. Among the mint extracts, the highest diversity of phenolic acids was found in the acetic macerates and tinctures. Moreover, the mint macerate was characterized by the largest diversity of phenolic compounds.

The largest amounts of phenolic acids were found in the lavender tincture, which was also characterized by the highest diversity of phenolic compounds of all the lavender extracts, while fermented vinegar had the lowest concentration.

In the case of rose petal extracts, the tinctures and macerates contained the highest amounts of flavonoids with the prevalence of kaempferol 3-*O*-galactoside, kaempferol 3-*O*-glucoside, and quercetin. The fermented vinegar was the poorest in phenolic compounds among the rose petal extracts. (Table 2).

The analysis of the metal concentration revealed significant differences between types of fruit and herbal extract (Table 3). In all plant materials, except rosehips, the highest concentration of calcium, magnesium, and aluminum was found in macerates. In the case of rosehips, the highest concentration of metals was found in decoctions. The concentrations of iron and zinc were also highest in macerates in comparison to other extract types, although the overall amounts were very small, such that the differences were not statistically significant. Arsenic, lead, and mercury were not detected in tested extracts and the results for those elements are not shown in Table 3.

### 3.2. Antioxidant Power

The antioxidant power values of the extracts measured with FRAP are presented in Table 4.

The strongest antioxidant abilities were demonstrated by all the rosehip extracts (in order from highest to lowest: tincture, decoction, macerate, and fermented vinegar). Then, tinctures from the remaining plants revealed high antioxidant power (in order: mint, raspberry, rose petals, and lavender). The fermented vinegars from lavender, apple peels, raspberry, and mint were characterized by very low antioxidant abilities. The lowest reduction power was found in the mint extracts (in order from the lowest to the highest: decoction, macerate, and fermented vinegar).

There was no correlation found between the antioxidant power of extracts and the metal concentrations.

**Figure 2:**
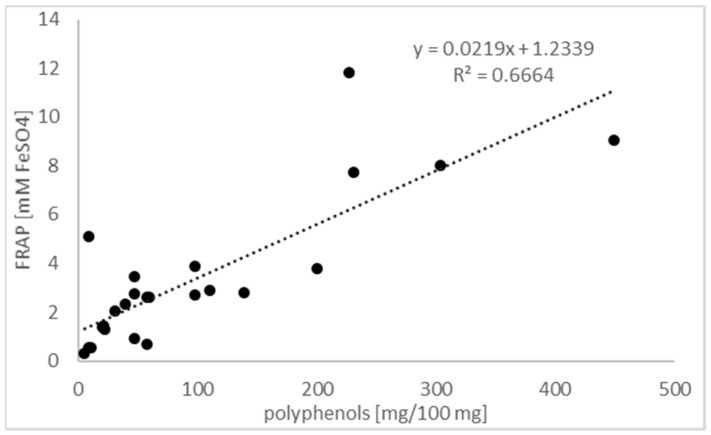
The relation between polyphenol concentration in the extracts analyzed using Fast Blue BB assay and antioxidant power measured with FRAP.

The FRAP values were correlated with total phenolic content (*y* = 0.0219*x* + 1.2339; *R*^2^ = 0.6664, *p* = 0.039) as shown in Figure 2.

### 3.3. Viability of Cells

The prepared fruit and herbal extracts were not found to be cytotoxic to human lymphocytes during 1 h of incubation at 37 °C (Table 5).

### 3.4. DNA Damage

**Figure 3:**
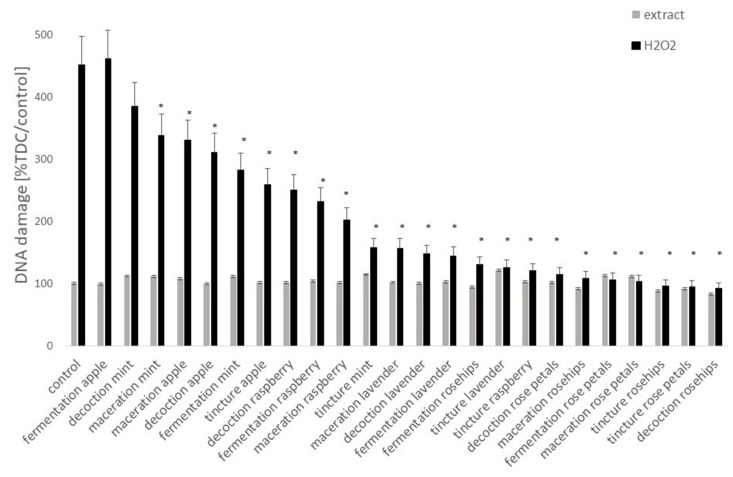
The DNA damage in lymphocytes measured using the comet assay after exposure to the analyzed extracts for 1 h. Oxidative DNA damage was induced by incubation of cells in 25 µM H_2_O_2_. * Asterisks indicate the significant difference between the results obtained for cell treated with plant extracts and H_2_O_2_ in comparison to cells treated with H_2_O_2_ only. The results of the comet assay are expressed as tail DNA content (TDC) and are presented as % of damages in control sample (untreated cells).

The comet assay revealed that none of the extracts caused damages to the DNA of lymphocytes (Figure 3) and analysis of variance confirmed no statistical differences between the samples exposed to plant extracts and the control sample (ANOVA *p* > 0.05 all cases).

The genoprotective effect of the vinegars, macerates, tinctures, and decoctions from fruit and herbs against DNA damage was analyzed on lymphocytes treated with hydrogen peroxide. The level of DNA fragmentation in the lymphocytes was reduced significantly in comparison with the cells exposed only to hydrogen peroxide (ANOVA *p* < 0.05) in all the samples except apple fermented vinegar and mint decoction, in which the differences were not statistically significant (Figure 3).

**Figure 4:**
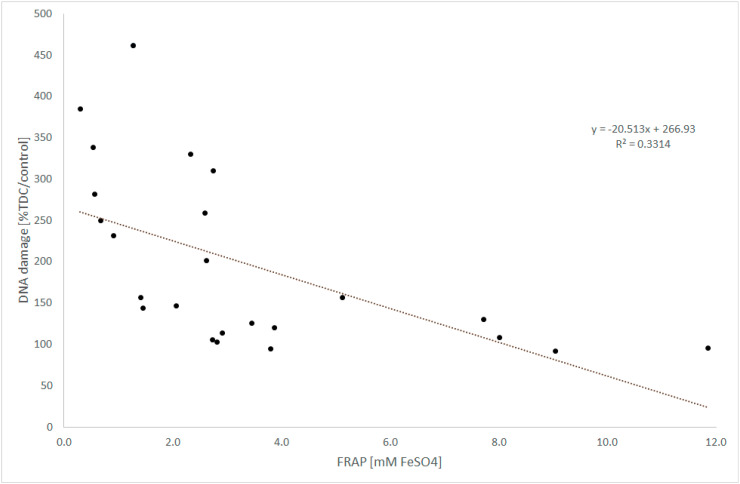
The relation between the genoprotective abilities of plant extracts and their antioxidant power. Lymphocytes were exposed to the extracts analyzed for 1 h. Oxidative DNA damage was induced by cell incubation in 25 µM H_2_O_2_ for 5 min. The results of the comet assay are expressed as tail DNA content (TDC) and are presented as % of control damages.

The protective effect of fruit and herbal extracts was correlated with antioxidant power (Figure 4) (*y* = −20.513*x* + 266.93; *R*^2^ = 0.3314; *p* = 0.02) as well as with the polyphenols’ concentration *y* = −0.5371*x* + 250.19; *R*^2^ = 0.3161; *p* = 0.034). The tinctures demonstrated the highest protective abilities, but fermented vinegars had the lowest.

The DNA damage level to the cell after the induced oxidative stress was negatively correlated with the concentration of polyphenols (*y* = −0.5371*x* + 250.19, *R*^2^ = 0.3161; *p* = 0.026) and the antioxidant properties of extracts *y* = −20.475*x* + 268.66; *R*^2^ = 0.3145; *p* = 0.0289) (Figure 4).

## 4. Discussion

The goal of this study was to compare fermented vinegars from edible fruits and aromatic herbs with other types of extracts traditionally prepared for culinary and medicinal purposes, such as: acetic macerates, decoctions, and tinctures, and as it had been assumed, these extracts differed significantly in terms of the content of the active ingredients analyzed. Differences could be observed between the plants used for extraction as well as between the types of extracts. Therefore, the claim that one extraction method is the most appropriate one for all herbs and fruits cannot be justified.

This study proves that rosehips are a plant material that is very rich in phytochemicals [25,26,27,28]. However, it is necessary to remember that the phytochemical content of fruits may vary according to many factors, such as the stage of ripening, weather, and regional climate [29,30,31]. Only rosehip extracts were found to be a good source of vitamin C, as 100 g of each rosehip extract could cover the recommended dietary allowances for vitamin C [32], which is consistent with the available data on the vitamin C content of rosehips [18,27,28]. The highest concentration of ascorbic acid was found in the rosehip decoction, which suggests that this would be the best way to preserve these fruits. Moreover, only the rosehip decoction contained detectable amounts of carotenoids at a concentration above 1 µg/100 g, although this value is still low in comparison with the daily recommended allowance, which is 800 µg/day [32].

The metal concentration analysis also revealed significant differences between the types of fruit and herbal extracts. The highest concentrations of metals were found in macerates, which may depend on the low pH of this type of extract and the fact that an acidic environment influences the plant material from the very beginning of the extraction process; thus, this process allows the longest interaction with cations. It was also observed that the aluminum content exceeded the maximum accepted level in water (60 µg/L) in raspberry extracts and all macerates and fermented vinegars from all plants except rosehips and rose petals. All the raspberry extracts contained high amounts of nickel exceeding the maximum level of this metal in water (50 µg/L) [33], which suggests that this fruit may be particularly prone to nickel accumulation [34]. Furthermore, all these extracts, except those prepared from rosehips, exceeded the permitted levels for chromium. As the extracts did not contain iron, magnesium, zinc, or calcium in high quantities, they cannot be considered to be a rich dietary source of those minerals. The relatively high calcium and magnesium content in the rosehip decoction proves that this method of fruit processing is the most suitable for rosehips.

These extracts differed strongly in terms of their antioxidant properties depending to their type and the plant they were prepared from, which is consistent with previous observations [21]. It is not possible to indicate clearly which extract type is the best for any plant material, because the extract composition depends on the material which was used for preparation. However, some regularities could be observed. First of all, the highest flavonoids concentration was found mostly in tinctures, which justifies the phytotherapeutic uses of this type of extracts. The tinctures are not only the rich source of flavonoids and anthocyanins, but, additionally, most of the tinctures were characterized by the highest antioxidant abilities. This confirms the results of previous studies indicating that the ethanolic extraction is a very efficient method to reveal the antioxidant properties of herbs and fruits [21]. Fermented vinegars and acetic macerates prepared from the same plants did not differ significantly in terms of their antioxidant power, which was low in those cases. Furthermore, the anthocyanins were present only in apple peels and raspberry extracts and fermented vinegars contained the lowest amounts, while tinctures contained the highest level of those compounds. Moreover, HPLC analysis revealed that of the analyzed extracts’ fermented vinegars had the least diverse phenolic content.

There may have been doubts that the higher concentration of metal ions in acidic extracts could disturb the measurement of antioxidant power; however, the statistical analysis did not confirm that. Moreover, due to mass spectrometry analysis, the oxidation state of metals present in extracts is unknown; thus, the possible interaction with reducing agents is unevaluated. Nevertheless, the antioxidant power was correlated with the phenolic content of extracts and influenced their genoprotective capacities. We confirmed that the plant extracts’ mechanism of action depends on their polyphenol content [1,21] and the acidity of the extract was not important in this context [35,36]. Apple peels, mint decoctions, and fermented vinegars were found to have the lowest genoprotective abilities, while rosehips and rose petal extracts were the most active genoprotective agents. The low rank of decoctions may depend on the thermolability of some antioxidants, while the low antioxidant capacities of fermented vinegars could be caused by oxygen accessibility during the whole extraction period (four weeks), which could be the reason for the oxidation of some phytochemicals or their consumption by growing microorganisms.

Food ingredients can modulate the risk of cancer initiation, hence the unabated need to supply antioxidants to the organism. Herbs and fruits have been of interest for many years as they are an important source of chemopreventive ingredients, such as polyphenols, especially flavonoids. Flavonoids have been an object of special interest in recent years due to their antioxidant properties and the great variety of studies suggests that consuming polyphenol-rich food decreases the risk of cancer [37,38,39,40,41]. Due to the antioxidant properties of flavonoids and other polyphenols, homemade extracts from fruits and herbs may act as effective genoprotective agents [18,21,39,41]. Our data are consistent with publications that describe roses as a good source of antioxidants, especially polyphenols [18,21,42,43,44], and as we have proven, their genoprotective properties strongly depend on their polyphenol content. Moreover, it has been proven that natural food products have a much stronger effect than supplements [45,46,47].

## 5. Conclusions

Preparing fermented vinegars from fruits and herbs is one of the possibilities of processing plant materials for culinary and pro-health purposes. The advantage of vinegars is the fact that they can provide active phytochemicals, while they do not contain alcohol or sugar and are low in calories [32]. Furthermore, they can be produced from leftovers such as fruit peels, in which case acetic fermentation follows a zero-waste trend. However, the very low pH of vinegars means that they can intensify negative sensations in the digestive system and tooth enamel softening [1,48]. Nevertheless, a typical use of vinegars is as an additive to foods or drinks, in this case they are used in small amounts, hence their low pH has a small impact on the body, even though the supply of plant compounds from vinegar is rather limited. Moreover, as it has been observed, the phytochemical composition of fermented vinegars is poorer in comparison with that of tinctures and decoctions which are commonly used both in traditional medicine and in cookery, as the food is the best medicine. Nonetheless, according to the ever-growing demand for phytochemicals and antioxidants [48,49,50,51,52], and in reference to Hippocrates words: “Let food be thy medicine, and let medicine be thy food”, it is advisable to include natural polyphenols in the daily diet in every possible way, including in the form of fermented vinegars. When consumers use vinegar in cooking, they can use fermented vinegar from fruits or herbs instead of spirit vinegar, because even small amount of flavonoids provided with fermented vinegars is still an added source of dietary antioxidants.

## Figures and Tables

**Table 1:** The polyphenols, vitamin C, and carotenoid content in fruit and herbal extracts.

Plant	Extract	pH ± S.D.	Polyphenols [mg/100 g] ± S.D.	Vitamin C [mg/100 g] ± S.D.	Carotenoids [µg/100 g] ± S.D.
	food vinegar	2.51 ± 0.13	0.56 ± 0.34	ND	0.01 ± 0.01
Apple peels	Fermented	2.93 ± 0.17	22.07 ± 1.84	ND	0.13 ± 0.08
	Macerate	2.51 ± 0.08	38.87 ± 0.98	ND	0.06 ± 0.03
	Tincture	4.72 ± 0.14	59.74 ± 2.67	ND	0.19 ± 0.06
	Decoction	4.11 ± 0.17	47.24 ± 1.54	ND	0.12 ± 0.08
Raspberries	Fermented	2.82 ± 0.19	46.44 ± 2.54	1.67 ± 1.16	0.23 ± 0.07
	Macerate	2.7 ± 0.13	56.98 ± 3.00	5.00 ± 1.67	0.54 ± 0.02
	Tincture	3.85 ± 0.01	97.57 ± 2.06	1.67 ± 1.00	0.35 ± 0.04
	Decoction	3.98 ± 0.14	57.48 ± 1.87	ND	0.57 ± 0.67
Rosehips	Fermented	2.85 ± 0.13	231.32 ± 8.09	88.33 ± 2.33	0.27 ± 0.02
	Macerate	2.67 ± 0.10	303.38 ± 6.67	118.33 ± 1.00	0.27 ± 0.03
	Tincture	4.23 ± 0.09	227.42 ± 6.11	141.67 ± 3.67	0.29 ± 0.11
	Decoction	4.91 ± 1.00	449.68 ± 9.33	256.67 ± 5.43	1.17 ± 0.12
Mint	Fermented	2.79 ± 0.13	8.94 ± 1.00	ND	0.04 ± 0.01
	Macerate	2.59 ± 0.05	10.68 ± 0.67	ND	0.18 ± 0.00
	Tincture	6.96 ± 0.11	8.90 ± 1.22	ND	0.03 ± 0.00
	Decoction	6.98 ± 1.06	4.76 ± 0.89	ND	0.31 ± 0.02
Lavender	Fermented	2.86 ± 0.30	20.84 ± 1.50	ND	0.13 ± 0.04
	Macerate	2.61 ± 0.18	20.29 ± 1.12	ND	0.06 ± 0.05
	Tincture	6.99 ± 0.02	46.63 ± 1.89	ND	ND
	Decoction	6.59 ± 0.16	30.10 ± 1.51	ND	0.17 ± 0.01
Rose petals	Fermented	2.93 ± 0.07	97.17 ± 1.45	ND	0.09 ± 0.01
	Macerate	2.78 ± 0.13	138.51 ± 3.99	ND	0.09 ± 0.00
	Tincture	6.87 ± 0.20	199.81 ± 3.33	ND	0.29 ± 0.01
	Decoction	6.77 ± 0.09	109.82 ± 4.67	ND	0.11 ± 0.01

ND—non-detectable; S.D.—Standard deviation.

**Table 2:** Flavonoids and phenolic acids identified in fruit and herb extracts analyzed with HPLC.

Plant	Apple Peels				Raspberries				Rosehips Flesh				Mint Leaves				Lavender Flowers				Rose Petals			
Extract	Fermented	Macerate	Tincture	Decoction	Fermented	Macerate	Tincture	Decoction	Fermented	Vinegar	Tincture	Decoction	Fermented	Macerate	Tincture	Decoction	Fermented	Macerate	Tincture	Decoction	Fermented	Macerate	Tincture	Decoction
PHENOLIC ACIDS	[mg/100mL]																							
caffeic acid													0.07 ± 0.04	0.38 ± 0.02	0.18 ± 0.00	0.06 ± 0.00	5.08 ± 0.22	2.98 ± 0.06	4.47 ± 0.14	3.71 ± 0.07				
chlorogenic acid	0.63 ± 0.00	0.77 ± 0.29	2.08 ± 0.13	0.82 ± 0.01					0.38 ± 0.02	0.43 ± 0.02	2.32 ± 0.19	1.65 ± 0.10	0.13 ± 0.04	0.17 ± 0.02		0.04 ± 0.00							7.23 ± 0.59	3.86 ± 0.16
cinnamic acid																		0.39 ± 0.02	0.59 ± 0.02	0.92 ± 0.01				
elagic acid					2.11 ± 0.07	3.76 ± 0.51	12.45 ± 1.05	4.31 ± 0.15		0.62 ± 0.07	2.21 ± 0.32	1.28 ± 0.05												
ferulic acid																	2.63 ± 0.12	3.13 ± 0.43	3.26 ± 0.12	3.48 ± 0.07				
isoferulic acid																	1.85 ± 0.01	2.03 ± 0.14	2.99 ± 0.22	2.73 ± 0.05				
gallic acid	0.24 ± 0.02	0.26 ± 0.03	0.10 ± 0.01	0.34 ± 0.03					3.09 ± 0.02	1.30 ± 0.02	3.71 ± 0.41	10.60 ± 0.32	0.12 ± 0.04	0.20 ± 0.01	0.04 ± 0.01	0.13 ± 0.01						0.41 ± 0.02	1.26 ± 1.16	4.53 ± 0.10
p-coumaric acid																					1.27 ± 0.02	0.59 ± 0.02	3.04 ± 0.29	1.31 ± 0.10
p-hydroxybenzoic acid													1.08 ± 0.01	1.09 ± 0.01										
protocatechuic acid	0.34 ± 0.01	0.37 ± 0.00	0.13 ± 0.00	0.39 ± 0.01	0.48 ± 0.07	0.95 ± 0.03	0.91 ± 0.04	1.04 ± 0.03																
rosmarinic acid														0.38 ± 0.01	0.70 ± 0.01	0.23 ± 0.01		1.73 ± 0.04	4.61 ± 0.33	1.33 ± 0.14				
vanillic acid													0.07 ± 0.04	0.08 ± 0.00			3.66 ± 0.12	2.70 ± 0.06	4.53 ± 0.05	3.63 ± 0.11		0.22 ± 0.01	0.89 ± 0.03	
PHENOLIC ACIDS SUM	1.21 ± 0.11	1.40 ± 0.03	2.32 ± 0.11	1.55 ± 0.03	2.59 ± 0.08	4.71 ± 0.11	13.36 ± 1.01	5.35 ± 0.16	3.47 ± 0.02	2.34 ± 0.08	8.24 ± 0.53	13.53 ± 0.33	1.46 ± 0.40	2.30 ± 0.07	0.93 ± 0.01	0.46 ± 0.01	13.22 ± 0.23	12.95 ± 0.46	20.44 ± 0.26	15.79 ± 0.21	1.27 ± 0.02	1.21 ± 0.02	12.41 ± 0.58	9.70 ± 0.15
FLAVONOIDS	[mg/100mL]																							
apigenin														2.03 ± 0.01	5.17 ± 0.18	2.45 ± 0.12			3.92 ± 0.22					
catechin									8.32 ± 0.13	12.04 ± 1.04	58.90 ± 0.69	47.20 ± 0.21	2.28 ± 0.40	3.46 ± 0.07	0.60 ± 0.03	1.98 ± 0.20						8.36 ± 0.52		2.76 ± 0.05
epicatechin	7.80 ± 0.55	8.99 ± 0.28	13.28 ± 0.45	9.49 ± 0.52																	7.03 ± 0.05	1.12 ± 0.04		
kaempferol						5.70 ± 0.05	6.64 ± 0.05	5.54 ± 0.08													3.06 ± 0.02	6.39 ± 0.06	6.23 ± 0.14	
kaempferol 3-*O*-galactoside					0.25 ± 0.01	1.57 ± 0.04	6.37 ± 0.21	0.89 ± 0.06														20.11 ± 1.69	11.63 ± 0.86	4.79 ± 0.03
kaempferol 3-*O*-glucoside						1.52 ± 0.08	2.33 ± 0.17	1.39 ± 0.08														8.46 ± 1.02	12.87 ± 0.86	5.88 ± 0.83
kaempferol 7-ramnoside						1.33 ± 0.02	1.29 ± 0.05	1.20 ± 0.02													2.99 ± 0.09	4.60 ± 0.22	9.49 ± 1.16	3.84 ± 0.15
luteolin																			0.63 ± 0.02					
isoquercetin	1.51 ± 0.12	0.54 ± 0.01	1.69 ± 0.04	0.71 ± 0.02													0.29 ± 0.08	0.57 ± 0.02	1.79 ± 0.04	0.92 ± 0.01				
quercetin	1.74 ± 0.09	1.95 ± 0.11	1.66 ± 0.02	1.83 ± 0.09		1.57 ± 0.04	2.54 ± 0.08	1.79 ± 0.00		1.29 ± 0.01	1.46 ± 0.05	1.36 ± 0.01							5.69 ± 0.04			7.72 ± 0.08	16.41 ± 0.36	6.28 ± 0.19
quercetin 3-*O*-arabinofuranoside	2.21 ± 0.22	3.69 ± 0.31	7.57 ± 0.02	4.06 ± 0.10																				
quercetin 3-*O*-glucuronide	7.63 ± 0.14	8.51 ± 0.15	19.85 ± 0.65	10.92 ± 0.32																				
quercetin 3-*O*-ramnoside	10.94 ± 0.69	12.86 ± 0.55	21.28 ± 2.48	11.02 ± 0.13																				
quercetin 7-*O*-glucoside					1.15 ± 0.02	1.14 ± 0.02	1.17 ± 0.04	1.13 ± 0.00													5.14 ± 0.33			
quercitrin									2.03 ± 0.02	4.37 ± 0.02	3.52 ± 0.06	2.97 ± 0.02							1.73 ± 0.04					
rutoside	1.82 ± 0.17	2.91 ± 0.14	5.70 ± 0.28	2.87 ± 0.04						0.77 ± 0.04	5.83 ± 0.08	3.40 ± 0.02		0.27 ± 0.02	0.23 ± 0.01				5.92 ± 0.25	1.56 ± 0.03		2.28 ± 0.11	4.55 ± 0.36	3.15 ± 0.04
FLAVONOIDS SUM	33.65 ± 0.95	39.46 ± 0.73	71.02 ± 2.62	40.91 ± 0.65	1.40 ± 0.02	12.83 ± 0.54	20.34 ± 1.10	11.92 ± 0.21	10.35 ± 0.21	18.47 ± 1.04	69.71 ± 0.90	54.93 ± 0.46	2.28 ± 0.40	5.75 ± 0.07	5.99 ± 0.20	4.43 ± 0.20	0.29 ± 0.08	0.57 ± 0.02	19.68 ± 0.26	2.49 ± 0.03	18.22 ± 0.36	59.05 ± 2.06	61.18 ± 1.89	26.69 ± 0.90
ANTHOCYANINS	[mg/100mL]																							
kuromanin		1.57 ± 0.00	2.03 ± 0.01	1.90 ± 0.00																				
idaein					2.23 ± 0.02	2.88 ± 0.01	7.14 ± 0.03	3.24 ± 0.00																
keracyanin					0.92 ± 0.04	1.44 ± 0.02	5.35 ± 0.03	2.42 ± 0.05																
ANTHOCYANINS SUM		1.57 ± 0.00	2.03 ± 0.01	1.90 ± 0.00	3.15 ± 0.04	4.32 ± 0.02	12.49 ± 0.03	5.66 ± 0.05																
TOTAL POLYPHENOLS	34.86 ± 0.96	42.43 ± 0.73	75.36 ± 2.62	44.35 ± 0.65	7.14 ± 0.08	21.86 ± 0.08	46.19 ± 0.55	22.93 ± 1.49	13.82 ± 0.26	20.81 ± 0.21	77.95 ± 1.04	68.46 ± 1.04	3.74 ± 0.57	8.05 ± 0.57	6.92 ± 0.10	4.89 ± 0.02	13.51 ± 0.20	13.52 ± 0.24	40.12 ± 0.46	18.28 ± 0.37	19.49 ± 0.21	60.26 ± 2.06	73.59 ± 1.98	36.38 ± 0.91

**Table 3:** Metal concentrations in fruit and herbal extracts analyzed using inductively coupled plasma mass spectrometry (ICP-MS).

Plant	Extract	Ca [mg/100 mL] ± S.D.	Fe [mg/100 mL] ± S.D.	Mg [mg/100 mL] ± S.D.	Zn [mg/100 mL] ± S.D.	Al [μg/L] ± S.D.	Ni [μg/L] ± S.D.	Cd [μg/L] ± S.D.	Cr [μg/L] ± S.D.
Apple peels	Fermented	1.50 ± 0.01	0.02 ± 0.00	2.45 ± 0.02	ND	68.1 ± 1.4	13.9 ± 1.1	0.3 ± 0.0	202.2 ± 12.2
	Macerate	5.10 ± 0.00	0.06 ± 0.00	2.31 ± 0.01	0.01 ± 0.00	80.2 ± 2.3	13.3 ± 1.0	0.3 ± 0.0	192.9 ± 10.9
	Tincture	0.35 ± 0.01	ND	1.55 ± 0.00	ND	34.2 ± 1.9	17.2 ± 1.5	ND	167.9 ± 11.6
	Decoction	1.02 ± 0.01	ND	1.77 ± 0.01	0.01 ± 0.00	33.1 ± 2.6	10.9 ± 1.0	0.1 ± 0.0	66.9 ± 6.6
Raspberries	Fermented	4.27 ± 0.01	0.04 ± 0.00	5.42 ± 0.00	0.03 ± 0.00	40.6 ± 2.1	62.7 ± 2.5	4.5 ± 0.0	68.1 ± 5.9
	Macerate	6.66 ± 0.02	0.04 ± 0.00	5.75 ± 0.00	0.06 ± 0.00	64.8 ± 3.4	63.2 ± 2.3	7.8 ± 0.1	133.1 ± 4.3
	Tincture	2.47 ± 0.00	0.01 ± 0.00	5.38 ± 0.00	0.07 ± 0.00	62.4 ± 2.8	103.2 ± 5.6	2.8 ± 0.0	184.8 ± 15.0
	Decoction	3.78 ± 0.00	0.01 ± 0.00	5.17 ± 0.02	0.07 ± 0.00	49.8 ± 5.8	73.9 ± 7.6	11.1 ± 0.8	158.8 ± 11.3
Rosehips	Fermented	ND	ND	ND	ND	1.0 ± 0.1	6.9 ± 1.1	ND	38.3 ± 4.0
	Macerate	ND	ND	ND	ND	1.1 ± 0.0	7.1 ± 0.6	ND	57.0 ± 1.9
	Tincture	0.01 ± 0.00	ND	0.02 ± 0.00	ND	5.4 ± 0.1	6.8 ± 0.4	ND	25.4 ± 1.0
	Decoction	20.43 ± 0.09	0.21 ± 0.00	16.52 ± 0.10	0.07 ± 0.00	61.0 ± 2.7	72.0 ± 3.2	0.2 ± 0.0	39.2 ± 1.1
Mint	Fermented	5.39 ± 0.01	0.04 ± 0.00	1.34 ± 0.00	0.02 ± 0.00	64.6 ± 4.6	11.9 ± 0.9	0.5 ± 0.0	138.5 ± 15.9
	Macerate	9.70 ± 0.04	0.21 ± 0.00	1.89 ± 0.01	0.04 ± 0.00	175.0 ± 7.8	12.9 ± 0.8	3.1 ± 0.1	157.1 ± 13.0
	Tincture	0.18 ± 0.00	ND	0.61 ± 0.00	0.01 ± 0.00	40.8 ± 3.9	10.0 ± 0.9	ND	167.5 ± 12.6
	Decoction	3.00 ± 0.00	ND	0.89 ± 0.00	0.01 ± 0.00	6.7 ± 2.4	6.6 ± 0.1	0.2 ± 0.0	93.5 ± 5.6
Lavender	Fermented	6.04 ± 0.01	0.08 ± 0.00	4.66 ± 0.01	0.05 ± 0.00	297.5 ± 11.9	18.3 ± 2.0	0.5 ± 0.0	133.0 ± 3.3
	Macerate	6.71 ± 0.00	0.09 ± 0.00	6.42 ± 0.02	0.06 ± 0.00	304.3 ± 9.8	20.8 ± 1.9	0.4 ± 0.0	162.9 ± 7.8
	Tincture	0.18 ± 0.00	ND	0.95 ± 0.00	0.01 ± 0.00	43.2 ± 3.6	23.2 ± 1.0	ND	190.7 ± 8.9
	Decoction	1.43 ± 0.00	0.01 ± 0.00	1.97 ± 0.00	0.01 ± 0.00	7.3 ± 3.8	8.3 ± 0.3	ND	119.1 ± 10.0
Rose petals	Fermented	ND	ND	ND	0.05 ± 0.00	ND	ND	0.3 ± 0.0	146.5 ± 7.9
	Macerate	3.17 ± 0.01	0.03 ± 0.00	1.72 ± 0.00	0.01 ± 0.00	25.6 ± 2.7	20.0 ± 0.4	ND	156.0 ± 8.0
	Tincture	0.27 ± 0.00	ND	1.01 ± 0.00	0.01 ± 0.00	23.3 ± 2.7	17.7 ± 0.2	ND	113.9 ± 4.6
	Decoction	0.27 ± 0.01	ND	1.02 ± 0.00	ND	24.9 ± 2.3	16.4 ± 1.1	ND	ND

ND—non-detectable; S.D.—Standard deviation.

**Table 4:** The antioxidant power of fruit and herbal extracts measured using ferric reducing antioxidant power (FRAP) expressed as molar concentration of equivalents of FeSO_4_.

	FRAP [mM FeSO_4_] ± S.D.			
	Extract Type			
Plant	Fermented Vinegar	Acetic Macerate	Tincture	Decoction
Apple peels	1.9 ± 0.9	2.3 ± 0.2	2.6 ± 0.3	2.7 ± 0.3
Raspberries	0.9 ± 0.9	2.6 ± 0.3	3.9 ± 0.3	0.7 ± 0.1
Rosehips flesh	7.7 ± 2.6	8.0 ± 0.9	11.8 ± 0.9	9.0 ± 0.8
Mint leaves	0.6 ± 0.3	0.5 ± 0.2	5.1 ± 0.5	0.3 ± 0.1
Lavender flower	1.5 ± 0.6	1.4 ± 0.1	3.4 ± 0.3	2.1 ± 0.3
Rose petals	2.7 ± 0.9	2.8 ± 0.3	3.8 ± 0.4	2.9 ± 0.4

**Table 5:** Viability of lymphocytes exposed to plant extracts for 1 h at 37 °C.

	Cell Viability [%Control] ± S.D.					
	Plant Material					
Extract	Apple	Raspberry	Rosehips	Mint	Lavender	Rose Petals
Fermentation	82 ± 3	91 ± 8	86 ± 3	79 ± 0	80 ± 0	73 ± 1
Maceration	88 ± 2	90 ± 4	106 ± 9	85 ± 1	79 ± 4	93 ± 2
Tinctures	103 ± 0	105 ± 2	107 ± 0	89 ± 0	91 ± 3	103 ± 0
Decoctions	99 ± 2	93 ± 1	98 ± 0	89 ± 0	93 ± 0	100 ± 6

S.D.—standard deviation.
